# Peripheral blood cell microRNA quantification during the first trimester predicts preeclampsia: Proof of concept

**DOI:** 10.1371/journal.pone.0190654

**Published:** 2018-01-02

**Authors:** Edward E. Winger, Jane L. Reed, Xuhuai Ji, Kypros Nicolaides

**Affiliations:** 1 Laboratory for Reproductive Medicine & Immunology, San Francisco, California, United States of America; 2 Stanford University, Human Immune Monitoring Center, Stanford, California, United States of America; 3 King’s College Hospital, Brixton, London, United Kingdom; University Hospital Basel, SWITZERLAND

## Abstract

**Objective:**

We investigated the capacity of microRNAs isolated from peripheral blood buffy coat collected late during the first trimester to predict preeclampsia.

**Study design:**

The cohort study comprised 48 pregnant women with the following pregnancy outcomes: 8 preeclampsia and 40 with normal delivery outcomes. Quantitative rtPCR was performed on a panel of 30 microRNAs from buffy coat samples drawn at a mean of 12.7±0.5 weeks gestation. MicroRNA Risk Scores were calculated and AUC-ROC calculations derived.

**Results:**

The AUC-ROC for preeclampsia risk was 0.91 (p<0.0001). When women with normal delivery and high-risk background (those with SLE/APS, chronic hypertension and/or Type 2 Diabetes) were compared to women who developed preeclampsia but with a normal risk background (without these mentioned risk factors), preeclampsia was still predicted with an AUC-ROC of 0.92 (p<0.0001).

**Conclusion:**

MicroRNA quantification of peripheral immune cell microRNA provides sensitive and specific prediction of preeclampsia in the first trimester of pregnant women. With this study, we extend the range during which disorders of the placental bed may be predicted from early to the end of the first trimester. This study confirms that buffy coat may be used as a sample preparation.

## Introduction

Preeclampsia affects at least 2–3% of all pregnancies and is a major cause of maternal and perinatal morbidity and mortality [1]. The condition is recognized clinically after 20 weeks of gestation with the new appearance of hypertension and proteinuria. In countries with limited access to medical care, it is estimated that the disorder is responsible annually for greater than 60,000 deaths worldwide [2]. In developed countries, therapeutic intervention is often concluded with early delivery. While this intervention protects the mother, it results in significant morbidity and mortality to the neonate [3]. Early diagnosis has been a goal permitting intervention at an early time point [4].

Preeclampsia is among the great obstetrical syndromes that include premature rupture of the membranes, intrauterine growth retardation, premature birth and miscarriage. Khong, Brosens and Romero have identified histologic features of inadequate spiral artery transformation that are shared by each of the great obstetrical syndromes [5, 6]. Malperfusion results in placental stress from the beginning of the second trimester. Investigators have identified shed placental components in the plasma that correlate with malperfusion. The shed placental components have been used as biomarkers, often in combination with ultrasound and history, as predictors of the development of the great obstetrical syndromes.

Sweeting et al. note that a diagnostic regimen performed during the same time window as NIPT (non-invasive prenatal testing utilizing plasma DNA) would provide clinicians with important information at the outset of pregnancy management [7]. Plasma has been studied extensively as a carrier of material shed from tumors allowing for their non-invasive assessment, the so-called “liquid biopsy” [8]. Likewise, quantification of biomarkers within plasma such as PLGF, sFLT-1 and sENG [9,10,11] have been applied to pregnancy outcome prediction during the second and third trimesters [12, 13]. However, these markers can only identify 35% of all preeclampsia and approximately 40% of preterm births, at false-positive rate of 10% [14, 15, 16]. None of these markers can predict preeclampsia during the first trimester when the process of maladaptive placentation is initiated [17, 18].

Romero et al. identify events within the placental bed that first affects the placenta during the second trimester [5]. They emphasize that inadequate remodeling of spiral arteries within the placental bed leads to placental malperfusion and consequent placental dysfunction after the initiation of intervillous blood flow at the end of the first trimester. Maternal immune cells present from the beginning of pregnancy appear to have both a direct role in this spiral artery remodeling and in subsequent guidance of trophoblast activity [7, 19]. Thus, successful spiral artery remodeling involves maternal immune cells in addition to the well-understood role of trophoblast.

Prediction of preeclampsia during the first trimester by assessment of shed placental components is impaired. Prior to the second trimester, the spiral arteries of the placental bed are plugged by trophoblasts. During the majority of the first trimester, the placenta relies on histiotrophic nutrition and exists in a state of physiologic hypoxia, a state shared by all placentas until weeks 11 through 14 of gestation. After this, the trophoblast plugs begin to dissipate [20] initiating blood flow, increasing oxygen levels, producing a mature, non-proliferative, invasive trophoblast phenotype [21]. Nevertheless, significant effort has been made at discovering serum-based biomarkers for first trimester prediction of preeclampsia [22]. However, we speculate that the placenta destined to healthy as well as compromised outcome experiences essentially the same hypoxic conditions while spiral arteries remain plugged. Thus, the placenta during the first trimester would not be expected to differentially shed biomarkers into the plasma. Quantification of peripheral blood cell microRNA may offer a new approach.

MicroRNA is a class of RNA species comprising a 22–24 base non-coding polynucleotide. They integrate disparate genetic elements into collaborative metabolic and signaling pathways. They form networks that supervise coordinated expression of mRNAs that guide and maintain cell identity and buffer cell systems against changing conditions. MicroRNA has attracted great interest in the diagnosis and monitoring of various conditions including cancer, autoimmune, inflammatory and neurologic diseases [23].

In our previous studies, we found that first trimester peripheral blood mononuclear cell (PBMC) microRNA provides sensitive and specific prediction of preeclampsia and preterm birth when sampled within a range of 4–11 weeks gestation [24, 25]. In our present study, we wished to widen our first trimester microRNA sample collection to include specimens collected at a later time-point and to demonstrate that buffy coat samples may also be used. Samples from a biorepository of buffy coat specimens maintained at King’s College London were used in contrast to the PBMC sample collection interrogated in our prior studies. The sample archive utilized was collected at a later time point, at a range of 11–13 weeks gestation. Together, we hoped to demonstrate that quantification of first trimester peripheral blood cell microRNA, prepared as either PBMCs or as buffy coat is predictive of various disorders involving the placental bed throughout the first trimester.

## Materials and methods

### Study population and consent

MicroRNA quantification was performed on maternal buffy coat blood specimens obtained between 11–13 weeks gestation from women with singleton pregnancies attending their routine first hospital visit in pregnancy. Patients represented a non-selected patient population within the patient catchment region around King’s College London Hospital. “Normal delivery” was defined as the delivery of a singleton, normal karyotype baby with the following pregnancy criteria: delivery at 38–42 weeks gestation, baby weight within the normal range for gestational age and maternal BMI <30. Preeclampsia was defined according to the guidelines of the International Society for the Study of Hypertension in Pregnancy [26]. See Table 1 for population details.

**Table 1:** Study population.

Patient history:	Preeclampsia	Normal delivery	p value
**#samples**	8 (2 early onset; 6 late onset)	40	
**Age (yrs) (mean ± S.D.)**	30.9±8.8	33.3±6.5	0.39
**Race**:			
**White (%)**	25% (2/8)	53% (21/40)	0.24
**Black (%)**	63% (5/8)	25% (10/40)	0.88
**Asian**	0% (0/8)	20% (8/40)	0.32
**Mixed/Other (%)**	13% (1/8)	3% (1/40)	0.31
**Previous livebirth**	50% (4/8)	48% (19/40)	1.00
**Previous preeclampsia**	25% (2/8)	0% (0/40)	0.02
**Diabetes**	0% (0/8)	5% (2/40)	1.00
**SLE/APS**	0% (0/8)	18% (7/40)	0.58
**BMI (mean±S.D.)**	25.1±2.9	24.1±2.8	0.36
**Smoking**	25% (2/8)	8% (3/40)	0.19
**ART conception method (%)**	13% (1/8)	5% (2/40)	0.43
**Gest. age at sampling (weeks) (mean ± S.D.)**	12.6±0.4	12.7±0.5	0.58
**Gest. age delivery (weeks) (mean ± S.D.)**	35.6±5.4	39.8±0.9	<0.0001
**Birthweight in grams (mean ± S.D.)**	2354±1263	3343±317	<0.0001

Venous blood was obtained from women who had given written informed consent to provide samples for research as approved by the National Research Ethics Service of the National Health Service. Blood was collected in EDTA Vacutainer tubes (Becton Dickinson UK Ltd, Oxfordshire, United Kingdom) and processed within 15 minutes of collection. The tubes were centrifuged at 2000g for 10 minutes. Buffy coat was collected by pipette aspiration guided visually and frozen immediately at -80°C without an RNA preservative, labeled with a unique patient identifier and maintained for up to nine years. Specimens were shipped from King's College London (KCL), UK directly to the Stanford Human Immune Monitoring Center (Stanford, California, USA) on dry ice where they remained blinded as to clinical outcome through testing, identified only by a unique identification number. The study was a retrospective analysis using clinical data from patient charts and specimens frozen and stored as buffy coat. Institutional Review Board approval for retrospective analysis of first trimester peripheral blood specimens was obtained (Western Institutional Review Board (WIRB), Study Number 1151255, Pro. Number 20142368). Patient identifying information was maintained in accordance with HIPAA requirements.

### Inclusion criteria

For study inclusion, all samples were from patients that met the following criteria: 1) index cycle between April 2006 to July 2014; 2) singleton delivery; 3) delivered baby with no obvious birth defects; 4) BMI <30; 5) blood sample available from 11–13 weeks gestation. The original study population included 82 patients comprising 15 patients developing preeclampsia and 67 patients with normal deliveries. A total of 34 samples were degraded and thereby unsuitable for analysis. Forty-eight samples remained for study inclusion: 40 normal deliveries and 8 preeclampsia.

### MicroRNA selection and analysis

Each of the samples was analyzed using a panel of 30 microRNAs previously selected by their differential expression on microarray analysis between patients destined to compromised pregnancy and healthy pregnancies from a total pool of 852 microRNAs (3.5%) [24]. These microRNAs included: hsa-miR-340-5p, -424-5p, -33a-5p, -7-5p, -1229, -1267, -671-3p, -1, -133b, -144-3p, -582-5p, -30e-3p, -199a-5p, -199b-5p, -210, -221-5p, -575, -301a-3p, -148a-3p, -193a-3p, -219-5p, -132, -513a-5p, -1244, -16, -146a, -155, -181a, -196a and -223. Samples were reverse transcribed and rtPCR performed according to the protocol in our previous studies [24, 25]. Quantification was recorded as the PCR Ct (Ct or Cycle threshold, values correspond inversely to the microRNA concentration where Ct represents the number of PCR amplification cycles required to reach a detection threshold).

### Assignment of samples to training and validation sets

The 48 samples successfully quantified were randomized to two groups of 24 samples using the group randomization software from the Medcalc statistics suite version 16.1.2 (Medcalc^®^ version16.1.2, Ostend, Belgium). A first set of 24 samples was designated the “training” set and a second set of 24 patient samples was designated the “validation” set. To provide more even distribution of delivery outcome between training and validation sets given the small sample size, samples from patients who had healthy deliveries and those who developed preeclampsia were separately randomized.

### Development of the microRNA scoring system using the training set

MicroRNA Ct values were analyzed by Medcalc^®^ software generating AUC-ROC (Area Under the Curve-Receiver Operating Characteristic) values, their associated p values and Youden Index J Associated Criterion values for each microRNA in the training set (Table 2). The Youden Index J Associated Criterion Value for each microRNA was used as its positive/negative cut-off. A panel of microRNAs was established that included only those microRNAs where p ≤ 0.05. The panel and calculated cut-off points were then applied to the validation set. Ct values less than the cut-off value for each of the microRNAs in the panel were assigned a score of “1”. The sums of the individual microRNA scores for each sample in the validation set were designated the “Risk Scores”. An AUC-ROC was calculated from the Risk Scores for the validation set.

**Table 2:** Defining the final microRNA panel and positive/negative cutoffs using the training set.

No.	Selected for Panel	MicroRNA	AUC-ROC	p value	Associated criterion value	95% Confidence interval	Sensitivity	Specificity	Sample size	Positive group	Negative group
**1**	x	miR1267	0.88	0.0001	≤11.54	≤5.8 to ≤14.8	75	95	23	4 (17%)	19 (83%)
**2**	x	miR148a	0.93	0.0001	≤26.15	≤17.8 to ≤26.2	100	86	9	2 (22%)	7 (78%)
**3**	x	miR196a	1.00	0.0001	≤26.75	≤16.2 to ≤26.8	100	100	6	2 (33%)	4 (67%)
**4**	x	miR33a	1.00	0.0001	≤24.32	≤23. 2 to ≤24.3	100	100	11	3 (28%)	8 (73%)
**5**	x	miR575	0.93	0.0001	≤25.89	≤22.5 to ≤25.9	100	78	12	3 (25%)	9 (75%)
**6**	x	miR582	1.00	0.0001	≤23.26	≤22.5 to ≤23.3	100	100	2	7 (78%)	9 (75%)
**7**	x	miR210	0.86	0.0173	≤16.09	≤8.6 to ≤18.8	67	100	17	3 (18%)	14 (82%)
**8**	x	miR16	0.78	0.0526	≤12.36	≤7. 1 to ≤12.4	100	55	24	4 (17%)	20 (83%)
**9**		miR1229	0.83	0.0833	≤24.11	≤17.0 to ≤29.8	67	100	7	3 (43%)	4 (57%)
**10**		miR223	0.76	0.1038	≤7.948	≤5.8 to ≤12.3	50	100	24	4 (17%)	20 (83%)
**11**		miR133b	0.78	0.1211	≤20.71	≤19.1 to ≤26.0	75	95	23	4 (17%)	19 (83%)
**12**		miR155	0.74	0.1271	≤13.37	≤6.6 to ≤17.9	50	94	21	4 (19%)	17 (81%)
**13**		miR146a	0.80	0.1611	≤13.47	≤10.6 to ≤18.1	67	100	16	3 (19%)	13 (81%)
**14**		miR181a	0.80	0.1611	≤22.15	≤16.5 to ≤27.5	67	100	16	3 (19%)	13 (81%)
**15**		miR301a	0.70	0.2394	≤23.51	≤21.9 to ≤25.3	75	75	24	4 (17%)	20 (83%)
**16**		miR340	0.75	0.2481	≤22.59	≤20.3 to ≤26.9	75	89	22	4 (18%)	18 (82%)
**17**		miR30e-3p	0.68	0.3487	≤7.60	≤6.0 to ≤18. 3	50	95	24	4 (17%)	20 (83%)
**18**		miR132	0.65	0.5178	≤20.73	≤10.0 to ≤22.1	50	93	19	4 (21%)	15 (79%)
**19**		miR1244	0.62	0.5569	≤21.33	≤15.1 to ≤27.1	50	89	23	4 (17%)	19 (82%)
**20**		miR671	0.65	0.5813	≤24.05	≤19.6 to ≤28.8	50	100	18	4 (22%)	14 (78%)
**21**		miR7-5p	0.58	0.7371	≤23.24	≤14.7 to ≤27.7	50	85	17	4 (24%)	13 (76%)
**22**		miR1	NA	NA	NA	NA	NA	NA	1	1	0
**23**		miR144-3p	NA	NA	NA	NA	NA	NA	2	2	0
**24**		miR193a-3p	NA	NA	NA	NA	NA	NA	3	2	1
**25**		miR199a	NA	NA	NA	NA	NA	NA	1	1	0
**26**		miR199b-5p	NA	NA	NA	NA	NA	NA	3	2	1
**27**		miR219	NA	NA	NA	NA	NA	NA	4	2	2
**28**		miR221	NA	NA	NA	NA	NA	NA	1	1	0
**29**		miR424	NA	NA	NA	NA	NA	NA	4	2	2
**30**		miR513	NA	NA	NA	NA	NA	NA	1	1	0

Using the 24-sample training set, an AUC-ROC was calculated by Medcalc^®^ software for each of the 30 microRNAs quantified by rtPCR. MicroRNA where the p value was ≤0.05 were included in the panel. “NA” represents microRNA for which an insufficient number of samples were available to generate a ROC curve by the Medcalc software. The panel was limited to eight microRNAs: miR1267, miR148a, miR196a, miR33a, miR575, miR582, miR210 and miR16. A cutoff value for each was concurrently determined as the Youden Index J Associated Criterion Value.

## Results

### Validation of panel and microRNA cutoffs

Eight microRNAs in which the p value was p ≤ 0.05 in the training set were included in the scoring panel. The panel of eight microRNAs included miR-1267, -148a, -196a, -33a, -575, -582, -210 and -16 with the associated cut-off points for each of the panel members (Table 2). Using the Risk Scores for the 24 patient samples in the validation set (20 normal delivery; 4 preeclampsia) an AUC-ROC of 0.89 was calculated (p<0.0001). The composition of the panel and corresponding cutoffs calculated from the training set were, therefore, deemed validated.

### Application of the 8 microRNA panel Risk Score to the 48 patient population

The panel and cutoff criteria were then applied to each of the 48 patients (Table 3). A ROC curve was calculated using microRNA Risk Scores. AUC-ROC for preeclampsia risk was 0.91 (p<0.0001). When women with normal delivery and high-risk background (those with SLE/APS, chronic hypertension and/or Type 2 Diabetes) were compared to women who developed preeclampsia but with a normal risk background (without these listed risk factors), preeclampsia was still predicted with an AUC-ROC of 0.92 (p<0.0001) (Table 4).

**Table 3:** Preeclampsia risk assessment using 8 microRNAs to calculate Risk Scores in 48 patients.

Patient#	miR1267 ≤11.5	miR148a ≤26.2	miR16 ≤12.4	miR196a ≤26.8	miR210 ≤16.1	miR33a ≤24.3	miR582 ≤23.3	miR575 ≤25.9	Risk Score	Delivery Outcome
1	17.8	32.8	12.8	32.9	18.3	44.4	31.2	32.4	0	Normal Hypert
2	13.7	31.9	12	38.2	23.4	41.9	35.9	37.8	1	Normal Hypert
3	15	24.3	9.3	29.2	17.6		35.3	36.8	2	Normal Hypert
4	14.7		17.5			37		33.6	0	Normal Hypert
5	14.4	31.1	13.1	32.6	22.2	35.7	29	28.9	0	Normal Hypert
6	16.5	31.3	11.2	31.5	18.6	39.4	30.4	37.6	1	Normal
7	8	25.5	9.1	33.3	18.3	30.8	31.5	22.7	4	Normal
8	15.7	28.7	11.6	29.8	18.7	37.9	27.9	29.5	1	Normal
9	14	26.6	10.4	34.5	20.6	40.9	33.4	26.6	1	Normal
10	11.8	27.5	11.9	32.8	19.5	30.3	29.6	27.3	1	Normal
11	8.5		14.8			30		26.2	1	Normal
12	22.8	39.7	14.7		22.3				0	Normal
13	17.9	34.9	15.8	42.2	34.7	31.6	35.6	29.1	0	Normal
14	11.8	29.3	11.3	34.2	17.8		28.1	30.2	1	Normal
15	12.2	27.5	11.7	28.2	19.4	29.7	27.4	24.4	2	Normal
16	16	31	12.4	31	19.4	43.4	29.9	31.2	0	Normal
17	15	28.8	10	36.8	18.6	40.2	38.3	36.6	1	Normal
18	15.1	33.4	13.3		40	36.4	37.3	28.7	0	Normal
19	16.1	37	12.7	42	22.2		42.9	43.3	0	Normal
20	19.4	30.6	14.2	37.6	28	34	31.1	29.8	0	Normal
21	15.9	33.1	13.5	34.9	20.4		31.2		0	Normal
22	13.7	26.4	9.9	32.2	17.7	33.9	32.9	29.1	1	Normal
23	14.2	35.6	13.1			33.8		32.4	0	Normal
24	21.4	32.7	11.3		32.4	25.8		30.6	1	Normal
25	15.8	36.3	14.8	40.3	24.4	40.9	39.5	32	0	Normal
26	15.1		14.5			28.4		28.9	0	Normal
27	12.4	32.6	11.6	33.6	22.2	34.8	40.6	25.6	2	Normal
28			13.4			25.1		28	0	Normal
29	20.3	36.8	13.8	38.5	19.4		34.2		0	Normal
30	17.7	33.4	14.1		34	32.8	35.6	30.9	0	Normal
31	16.3	26.6	9.9	31	19.7	28.9	30.6	34	1	Normal
32	17.1	27.9	12.2	36.1	21.5		34.7	39.1	1	Normal APS/SLE
33	31	37	15.2	35.8	26.5	38.9			0	Normal APS/SLE
34	14.3	35.8	14.3		37	30.1	36	27	0	Normal APS/SLE
35	13.3	35.6	10.9	35.6	19.9			36	1	Normal APS/SLE
36	19	32.8	15.6	34.4	26.1	28	29.1	26.4	0	Normal APS/SLE
37	16.6	29.3	12.1	38.1	21.3		36.9	31.5	1	Normal APS/SLE
38	13.9	28.8	10.7	40.8	20		36.9	32.6	1	Normal APS/SLE
39	11.6	35.8	12.4		27.2				0	Normal T2DM
40	12.3	27.7	9.8	35.8	19.1		34.9	36.1	1	Normal T2DM
41	14.8	34.6	12	32.8	18.8	39.8	31.7	32.3	1	Preeclampsia
42	13.4	26.9	8	34.3	15.3		31.3	29.5	2	Preeclampsia
43	11.5		12.4			23.3		25.9	3	Preeclampsia
44	9.3	22.3	7	23.1	16	23.1	28.5	20	7	Preeclampsia
45	10.8	26.2	7.1	26.8	16.1	24.3	23.3	22.5	8	Preeclampsia
46	20.9	25.4	11.4	31.9	18.9		34.3	34.4	2	Preeclampsia
47	5.8	17.8		16.2	8.6	16.4	22.5	15	7	Preeclampsia
48	17.6	31.3	11	36.9	19.5		36.2		1	Preeclampsia

MicroRNA PCR Ct cut-off values for eight microRNAs were applied to each patient (designated in rows). A point was given for each microRNA level where the result was less than the threshold value (colored boxes). The points for each sample are summed as the “Risk Score”.

**Table 4:** AUC-ROC calculations for preeclampsia study populations.

	Preeclampsia vs. No preeclampsia	Preeclampsia vs. High Risk Normal Delivery (SLE/APS, T2DM and/or Chr. Hypert.)
**Column**	A	B
**No. Samples**	48	22
**AUC-ROC**	0.91	0.92
**Positive group**	8	8
**Negative group**	40	14
**Sensitivity**	75	75
**Specificity**	90	93
**P value**	p<0.0001	p<0.0001

An AUC-ROC curve analysis was performed using the Risk Scores for preeclampsia prediction.

## Discussion

Our study is the first successfully to use microRNA in peripheral blood to predict preeclampsia outcome using buffy coat specimens during the first trimester. Our present data extend our previous findings in several ways. First, we have shown that we can identify patients suffering preeclampsia risk from normal risk population when sampled at a mean of 12.7±0.5 weeks gestation. Second, we have shown that we can distinguish preeclampsia and a high-risk population comprising autoimmune disease, type 2 diabetes or chronic hypertension (Table 4, column B). Finally, samples were prepared as buffy coats rather than as PBMCs as in our previous studies demonstrating that buffy coat specimens are suitable for assessment.

Our current study is supported by our previous published findings [24, 25, 27]. Samples were collected at various time-points through the first trimester. The same analytic methods were used throughout the four studies and provided consistent results. AUC-ROC values were previously calculated at 0.90 or greater for preeclampsia, preterm birth and miscarriage using PBMC-prepared specimens [24, 25, 27].

Several members of the microRNA panel that we found predictive of preeclampsia risk may have functions associated with preeclampsia’s pathogenesis. For example, miR-210 can down regulate PTPN2 mRNA contributing to the pathogenesis of preeclampsia and has a central role in the switch from trophoblast proliferation to an invasive phenotype [28]. MicroRNA-148a has been shown to downregulate HLA-G expression which may prevent inhibition of NK cell killing through the LILRB1 inhibitory receptor [29]. MicroRNA-582-3p has been demonstrated to suppress EG-VEGF expression and inhibit trophoblast invasion and migration. From this, we are tempted to speculate that decidual immune cells may have a unique role prior to the recruitment of trophoblast to the spiral artery during the post-implantation period [30].

Immune cells within the decidua participate in the regulation of immune tolerance as well as providing essential support in the remodeling of spiral arteries within the placental bed [31]. Interrogation of immune cells within the decidua might provide predictive information about the future course of a pregnancy. However, acquisition of the decidual tissue is invasive, limiting any such immune investigation to selected, high risk patients. Our studies quantifying microRNA within peripheral blood cells during the first trimester offer a promising alternative [24, 25, 27].

The study had some limitations. Because of the small sample set, following validation, the criteria established with the training set were then applied to the combined data set introducing potential overfitting of the data. A larger population size is needed to confirm clinical utility. Also, most of the buffy coat specimens were frozen for many years without an RNA preservative.

In conclusion, our data suggest that the identification of a maternal immune cell surrogate provides a promising new path to early prediction and intervention. While permitting concurrent assessment along with NIPT, such a tool would also permit assessment of pregnancy risk both in the first trimester in the obstetric clinic and following embryo transfer at IVF clinic. As therapies directed to prevention of the various disorders of the placental bed become available, earlier intervention and treatment becomes possible. We suggest further studies to confirm and extend our findings.

## Supporting information

S1 File Supporting information file containing patient data and microRNA Ct expression levels. (XLSX)
